# Isolated blast crisis relapse in the central nervous system of a patient treating for a chronic myelogenous leukemia

**DOI:** 10.11604/pamj.2020.36.142.24155

**Published:** 2020-06-30

**Authors:** Houda Boudiaf, Khedaoudj Ezziane, Nassiba Ould Rouis, Meriem Himrane, Saliha Hakem, Hanifa Benchabane, Houria Boukhelal, Radja Arous, Lynda Chikhi

**Affiliations:** 1 Department of Pediatric Oncology, University Hospital of Mustapha Pacha, Algiers, Algeria,; 2 Department of Blood Transfusion and Hemobiology, University Hospital of Mustapha Pacha, Algiers, Algeria

**Keywords:** Chronic myeloid leukemia, central nervous system, blast crisis, imatinib

## Abstract

Chronic myeloid leukemia (CML) is a myeloproliferative disorder associated with the Philadelphia chromosome t (9;22) and the BCR-ABL fusion gene. The condition is relatively rare, accounting for 2.0% to 3.0% of childhood leukemia cases. CML has historically been a triphasic disease. Most patients are diagnosed in chronic phase. Without treatment, it inevitably progresses into a more aggressive accelerated phase and blast crisis. Some proportion of CML cases of blastic transformation develop an extramedullary disease that involves rarely central nervous system. This report describe an extremely rare case of 13-year-old girl with CML and extramedullary blast crisis in the central nervous system. Treatment options and monitoring of disease response are discussed.

## Introduction

Chronic myeloid leukemia (CML) is a myeloproliferative disorder associated with the Philadelphia chromosome t (9;22) and the *BCR-ABL* fusion gene. The treatment landscape has changed drastically over the last 17 years since the introduction of the tyrosine kinase inhibitor (TKI) imatinib [1,2]. CML in children is usually considered to be rare, but it accounts for 10% to 15% of myeloid leukemia. CML has historically been a triphasic disease. Approximately 85 to 90% of patients are diagnosed in chronic phase. Without treatment, it inevitably progresses to a more aggressive, accelerated phase and blast crisis. Majority cases of blastic transformation is characterized by increased blast cells in bone marrow and blood, however some proportion of CML cases develop extramedullary disease which involves lymph nodes, skin, soft tissues, serosal surfaces, bone, gastrointestinal, genitourinary tract [3-5] and rarely central nervous system (CNS) [6,7]. In our case report, we present a patient with CML in its blastic phase that developed in the central nervous system without evidence of disease in the peripheral blood (PB) and bone marrow (BM).

## Patient and observation

A 12-year-old girl presented an abdominal pain, high persistent fever of (40°C) and severe fatigue. The physical exam revealed pallor, hepatomegaly, and grade III splenomegaly. The investigation revealed an elevated white blood cell count (WBC): 545,000/mm^3^. PNN: 49,050/mm^3^, hemoglobin: 11.6g/dl, platelets: 328,000/mm^3^. The patient's peripheral blood smear revealed marked leukocytosis with a significant number of immature myeloid precursors and 21% blasts. The analysis of the BCR-ABL shows 94% in the peripheral blood. The patient was diagnosed with CML. She received a chemotherapy protocol made of hyperalkalinisation, allopurinol, hydroxyurea and a treatment based on imatinib at the dose of 400mg per day. Patient achieved complete hematological remission at three months and complete cytogenetic remission at 12 months. The patient was readmitted into our pediatric department with complains of severe headache, vomiting, pain and bilateral visual loss since three days. On examination, patient was afebrile and appeared pale. Blood pressure was 100/60mm/hg and the heart rate 98 beat/minute. There was no lymphadenopathy and the nervous system examination was normal. Intraocular muscle testing was normal; however, there was an exophthalmia in both eyes. The pupil was sluggish to react to light. Fundoscopy revealed bilateral stade III disc edema and hemorrhages of the retina.

**Figure 1:**
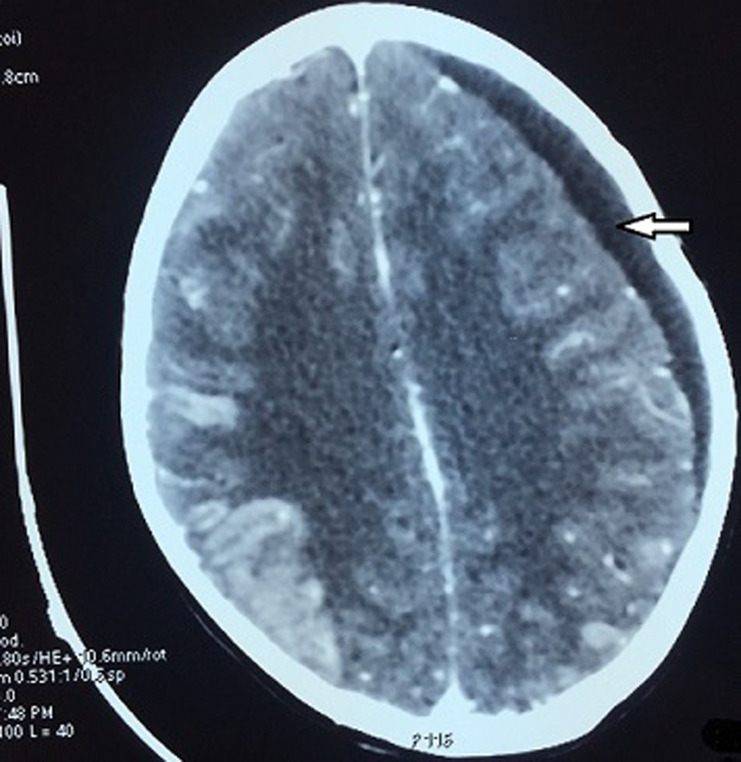
CT of the brain showing subdural chronic hematoma and a serpenginous gyriform enhancement around the contusion in the right parietal and occipital lobes

**Figure 2:**
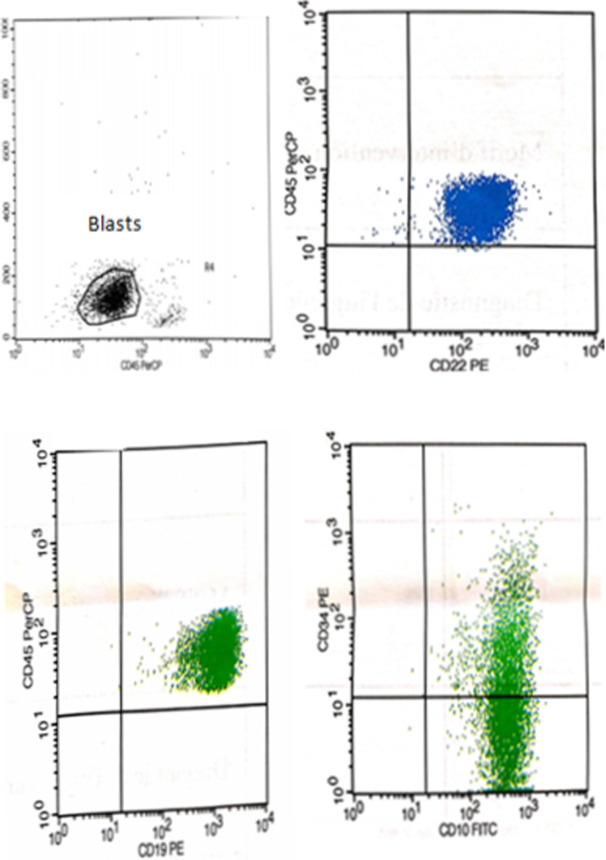
CSF flow cytometry showing expression of CD 10, CD19, CD22 on blasts

She underwent a brain computed tomography (CT) scan with the suspicion of leukemic involvement or any other intracranial event (cerebral edema); however, nothing was found. A subsequent bone marrow exam showed no morphologic or molecular evidence of CML or acute leukemia. Cytological evaluation of cerebrospinal fluid (CSF) was also negative. The patient had no other known pathology that could be held responsible for retina edema. We suggested that it was a side effect of imatinib so its administration was temporary interrupted. The patient was put on an anti-edema therapy (mannitol, dexamethasone). However, few months later, the patient presented in the emergency room with a headache and severe vomiting. She described a recurrence of the same character of headaches accompanied with weakness of extremities and seizures. Nervous system examination revealed neck rigidity, presence of meningeal signs and lower limb paraplegia. CT of the brain revealed subdural chronic hematoma and a serpenginous gyriform enhancement around the contusion in the right parietal and occipital lobes (Figure 1). Analysis of the CSF collected showed lymphoblasts in a WBC count of 11000cells/ml. Flow cytometry of the CSF showed that blast cells were positive for a cluster of differentiation markers (CD) (CD34, CD19, CD10, CD22 and partially positive for CD45) confirming CNS extramedullary lymphoid blast infiltration (Figure 2).

A bone marrow aspiration was done to evaluate the status of her chronic myelogenous leukemia and result was consistent with a chronic phase. Fundus examination showed bilateral optic atrophy. The diagnosis of extramedullary isolated CNS blast crises (lymphoid type) was based on the presence of blasts in CSF (confirmed by flow cytometry). The patient received a high-dose systemic induction chemotherapy and intrathecal therapy (methotrexate, arabinoside and dexamethasone). The imatinib was replaced by dasatinib at the dose of 100 mg per day. She was treated with 24 Gy of whole-brain radiation therapy. Allogenic stem cell transplantation was not feasible. We noticed a rapidly progressive amelioration in her neurological status after finishing systemic chemotherapy and physical therapy; however, the patient´s visual loss showed no signs of improvement.

## Discussion

Childhood chronic myeloid leukemia (CCML) is a malignant disease of granulocyte abnormal hyperplasia that is caused by clonal proliferation of pluripotent stem cells. It rarely occurs in children and typically evolves in three clinical stages: chronic, accelerated and blast phases. Blast phase of CML is defined either by the presence of more than 20% blasts in the peripheral blood/bone marrow or by the focal accumulation of blasts in the extramedullary sites in 5-10% of cases [8]. Lymph nodes, skin and soft tissues, bone, spleen are the commonest sites of extramedullary blast crises. However, isolated CNS blast crisis is uncommon and is limited to occasional case reports in adults or exceptional in children patients receiving imatinib mesylate treatment [9,10]. CNS blast crisis is usually presented with the clinical and radiological features of encephalitis and/or meningitis, which includes symptoms such as headache, cognitive changes, raised intracranial pressure and visual disturbances. The CSF is found positive for myeloid or lymphoid blasts and in some cases, molecular testing of the CSF revealed the typical BCR-ABL oncogene [11].

In our case, the diagnosis was delayed. A chronic benign headache was the first manifestation that was attributed to possible cerebral edema. Furthermore, it appears that repeated exams (lumbar puncture, brain imaging study) may sometimes be warranted as it increases the chances of identification of tumor cells. Imatinib mesylate is a potent and selective inhibitor of BCR ABL tyrosine kinase and the treatment of choice of CML in its chronic phase. It has also shown activity in the accelerated and blastic phases of CML. However, several studies [12,13] have shown that the penetration of the drug and its metabolites into the CNS is poor. Second-generation targeted BCR/ABL1 tyrosine kinase inhibitors, such as nilotinib and dasatinib, have an improved penetration of the blood-brain barrier. Porkka and colleagues reported the effectiveness of dasatinib therapy in CNS relapse in a CML rat model [14]. There is no standard recommendation of treatment CML involving the CNS. Agressive strategies (systemic and intrathecal chemotherapy, radiation and allogenic stem cell transplantation) are potential therapeutic modalities. Most of the reported cases were treated with combined intrathecal chemotherapy (variable combination of methotrexate, cytarabine, dexamethasone) and craniospinal irradiation. Our case was succesfully treated with intrathecal chemotherapy, cranial irradiation and a second-generation targeted BCR/ABL1 tyrosine kinase inhibitor (dasatinib). Allogeneic stem cell transplant, however, is the only modality that has been shown to lead to cure of this condition [15,16].

## Conclusion

The CNS, as a site of extramedullary blast crisis, is extremely rare in pediatric population. To our knowledge, there is no case reported yet in Algeria regarding pediatric CML in its blastic phase presenting only in the CNS. It is important to consider CNS relapse in chronic phase of CML patients treated with imatinib, especially those presenting a neurological symptoms such as headache, vision problems and lower limb´s paraplegic. This case emphasizes the need for brain imaging study and CSF monitoring in imatinib treated CML patients.
